# Prospect theory and tax evasion: a reconsideration of the Yitzhaki puzzle

**DOI:** 10.1007/s11238-016-9581-9

**Published:** 2016-11-14

**Authors:** Amedeo Piolatto, Matthew D. Rablen

**Affiliations:** 1 Barcelona Economics Institute (IEB), University of Barcelona, c/ J. M. Keynes, 1-11, 08034 Barcelona, Spain; 2 Department of Economics, University of Sheffield, 9 Mappin Street, Sheffield, S1 4DT UK

**Keywords:** Prospect theory, Tax evasion, Yitzhaki puzzle, Stigma, Diminishing sensitivity, Reference dependence, Variable audit probability, Endogenous reference level, H26, D81, K42

## Abstract

The standard expected utility (EUT) model of tax evasion predicts that evasion is decreasing in the marginal tax rate (the Yitzhaki puzzle). Recent literature shows cases in which incorporating prospect theory (PT) does and does not overturn the Puzzle. In a general environment that nests both PT and EUT preferences, we provide a detailed study of how the elements of PT affect the Puzzle. PT does not always reverse the Puzzle, hence we give and interpret conditions for when it does and does not. When allowing for stigma and/or variable audit probability, PT reverses the Puzzle in the same way and with the same limitations as does EUT, if equally augmented.

## Introduction

The expected utility theory (EUT) model of tax evasion predicts a negative relationship between tax rates and evasion whenever fines are imposed on the evaded tax and taxpayers exhibit decreasing absolute risk aversion (Yitzhaki 1974). Although empirical evidence on this question is mixed, and depends to an extent on the econometric methodology used (Bernasconi et al. 2014), a substantial body of empirical and experimental evidence finds a positive relationship between evasion and the tax rate (see, e.g., Ali et al. 2001; Alm et al. 1995; Clotfelter 1983; Crane and Nourzad 1986; Friedland et al. 1978; Pommerehne and Weck-Hannemann 1996; Poterba 1987).^1^ Owing to the weight of contradicting empirical evidence, and its counter-intuitive nature, the negative relationship between tax rates and evasion predicted by the EUT model has sometimes been termed the “Yitzhaki paradox” or “Yitzhaki puzzle” .

Prospect Theory (PT) has become a centrepiece of behavioural economics, for it is able to resolve many puzzles associated with EUT and it provides a better fit to much empirical data (Bruhin et al. 2010).^2^ It remains disputed, however, whether the application of PT to tax evasion resolves the Yitzhaki puzzle: Bernasconi and Zanardi (2004), Dhami and al-Nowaihi (2007) and Yaniv (1999), among others, each illustrate a specification of PT that resolves the Yitzhaki puzzle, but Hashimzade et al. (2013, p. 16) show alternative specifications that do not. Unpicking these divergent results is far from straightforward, however, as the elements of PT are specified differently across studies and as some studies invoke auxiliary assumptions (in addition to those of PT). Hashimzade et al. (2013) do consider some general specifications of PT, but do not directly compare to other preference models, while Dhami and al-Nowaihi (2007) undertake a comparison of PT and EUT, but not in a unified framework that nests EUT and PT, and for only one specification of PT. In this paper, therefore, we seek to evaluate, in a general sense, the marginal contribution of the elements of PT—individually and collectively—towards resolving the Yitzhaki puzzle. By disentangling the separate driving forces, we are able to reconcile seemingly contradictory results in the literature and to clarify which of the elements of PT, if any, contribute to solving the Puzzle. As these elements are now widely applied in the broader literature on behavioural decision making, by isolating the different components, our results can be readily extended to many further behavioural models besides PT.

We perform our evaluation in a general environment—which we shall term the Taxpayer behavioural model (TBM)—in which it is possible to vary (i) the specification of reference income; (ii) the elements of PT that are assumed to hold—to separate out the distinct effects of reference dependence, diminishing sensitivity, loss aversion, and probability weighting; (iii) the auxiliary assumptions assumed to hold vis-à-vis those of the standard portfolio model of tax compliance. The TBM is sufficiently general to encompass much of the existing literature, but sufficiently specific to yield conditions with clear economic and psychological interpretation.^3^

Our first contribution is to show that several seemingly disparate approaches to the specification of reference income in the existing literature are variants of a simple, yet general, formulation. Within this general formulation, our two main results are as follows: first, matching Yitzhaki’s original demonstration of the Puzzle under EUT, we give apparently plausible conditions under which the Puzzle still holds under PT (and stripped-down variants). Second, we find that, although some specifications of PT do reverse the Puzzle, such reversals often rely on the psychologically questionable implication that a tax rise makes taxpayers feel subjectively richer (relative to reference income) in the not-caught state, and in expectation. In particular, for PT to resolve the Puzzle, this condition *must* hold when preferences are homogeneous, a common assumption in applications of PT. Thus, while our results do not necessarily endorse the descriptive validity of EUT, we find nonetheless that a set of specifications of PT—which includes many specifications proposed in the literature—is either psychologically questionable, or share similar descriptive deficiencies in respect of the Puzzle.

We examine the implications for both EUT and PT (among other variants) of allowing for two auxiliary assumptions: social stigma costs and a variable audit probability. Allowing for sufficient social stigma always resolves the Puzzle under EUT, but not always under PT. In contrast, allowing for a variable audit probability does not clearly improve the ability of either EUT or PT to resolve the Puzzle. In general, the conditions under which these auxiliary assumptions improve the predictions of PT with respect to the Puzzle are the same as those which also improve the predictions of EUT.

By allowing for stripped-down variants of PT, we observe the marginal contribution of each of its elements. Under PT preferences, reference dependence is necessary to overturn the Yitzhaki puzzle, but the remaining PT elements—diminishing sensitivity, loss aversion and probability weighting—are neither necessary nor sufficient. These findings seem consistent with the nascent literature on the relative economic importance of the PT elements—reference dependence being the most widely accepted (see, e.g., Barberis 2013; Santos-Pinto et al. 2015).

The results of this study contribute to the literature on the use of non-expected utility preferences (and PT in particular) to explain tax evasion, and to the wider literature on the descriptive usefulness of non-expected utility preferences (Kim 2005; Harrison and Rutström 2009; Bruhin et al. 2010; Isoni 2011; Rees-Jones 2014; Masatlioglu and Raymond 2016). We do not claim that EUT is descriptively superior or inferior to PT over the full gamut of empirical regularities on tax-related behaviour, and other evidence relating to behaviour in risky settings more generally. Our results do, though, lead us to question the claim that PT is inherently better able to reconcile the Yitzhaki puzzle than is EUT.

The rest of the paper is organised as follows. Section 2 introduces a general model of the tax evasion decision that nests both PT and EUT preferences. In Sect. 3, we analyse the model for a fixed audit probability, and then for a variable audit probability. Section 4 concludes with a discussion of our findings and some wider thoughts on the choice of reference income and of preferences in applications of PT to tax evasion. All proofs are given in the Appendix.

## Taxpayer behavioural model

We now present a general model of the taxpayer tax evasion decision—the Taxpayer Behavioural Model—which nests PT, EUT and intermediate variants that may be considered as stripped-down versions of PT. Consider a taxpayer with an exogenous taxable income $Y>0$ (which is known to the taxpayer but not to the tax authority).^4^ The government levies a proportional income tax at marginal rate $t\in \left( 0,1\right)$ on declared income $X\in \left( 0,Y\right)$. The probability of audit is given by $p=p\left( X\right) \in \left( 0,1\right)$ with $p^{\prime }\le 0$, though taxpayers may behave as if they transform this objective probability into a decision weight $w\left( p\right)$, where $w\left( 0\right) =0$, $w\left( 1\right) =1$ and $w^{\prime }>0$.^5^ In the special case $p^{\prime }=0$ the probability of audit is independent, or “exogenous”, of declared income. Audited taxpayers face a fine at rate $f\in \left( 1,t^{-1}\right)$ on all undeclared tax, where the upper bound ensures that the amount paid (tax plus fines) never exceeds a taxpayer’s total income (hence limited liability is never violated, regardless of earned and declared income). Following Dhami and al-Nowaihi (2007), taxpayers may additionally incur social stigma equivalent to a monetary cost $s\left[ Y-X\right]$, where $s\ge 0$. Accordingly, $Y^{n}\equiv Y-tX$ is the taxpayer’s income when not caught, and $Y^{c}\equiv Y^{n}-\left[ tf+s\right] \left[ Y-X\right]$ is the taxpayer’s income when caught (audited).

The carrier of utility, *v*, is constructed to be sufficiently general to nest both EUT and PT preferences. Specifically, we first define a function *u* :  ${\mathbb {R}}\mapsto {\mathbb {R}}$ satisfying

A0. $u\left( x\right)$ is continuous and twice differentiable for all $x\in {\mathbb {R}}$, except possibly at $x=0$.
A1. $$u\left( 0\right) =0$$;
A2. $$u^{\prime }>0$$;
A3. $$u^{\prime \prime }<0$$;
A4. $$\partial A^{u}\left( x\right) /\partial x\le 0$$, where $$ A^{u}\left( x\right) \equiv -u^{\prime \prime }\left( x\right) /u^{\prime }\left( x\right) $$.^6^

The carrier of utility, *v*, is then defined as

1 $$\begin{aligned} v\left( x\right) =\left\{ \begin{array}{ll} u\left( x\right) &{} \quad \text { if }\;x\ge 0; \\ \left[ -1\right] ^{DS}\lambda u\left( \left[ -1\right] ^{DS}x\right) &{} \quad \text { otherwise}; \end{array} \right. \end{aligned}$$

where $$\lambda \ge 1$$, and the condition *DS* is true ($$DS=1$$) when diminishing sensitivity is assumed to hold, and false otherwise ($$DS=0$$). Note, first, that setting $$DS=0$$ and $$\lambda =1$$ we have $$v\left( x\right) =u\left( x\right) $$ as under EUT. In this case, A2 and A3 imply that *v* is increasing and concave. A4 implies that absolute risk aversion with respect to *v* is decreasing, although we note that for $$w\left( p\right) \ne p$$ risk preferences (in the sense of preferences for mean-preserving spreads) are determined jointly with respect to *v* and to *w* (Schmidt and Zank 2008).^7^ Note, second, that under diminishing sensitivity utility becomes $$ v\left( x\right) =-\lambda u\left( -x\right) $$ for $$x<0$$. In this case, A2 still implies that *v* is monotonic, but A3 implies that *v* is convex for $$ x<0$$. Following Köbberling and Wakker (2005), loss aversion with respect to *v* requires $$\lim \nolimits _{x\uparrow 0}\partial v(x)/\partial x>\lim \nolimits _{x\downarrow 0}\partial v(x)/\partial x$$, which holds if and only if $$\lambda >1$$. Assumption A4 implies with respect to *v* that

$$\begin{aligned} A\left( x\right) \equiv A^{v}\left( x\right) \equiv -\frac{v^{\prime \prime }\left( x\right) }{v^{\prime }\left( x\right) }\left\{ \begin{array}{ll}<0 &{} \quad \text { if } \; x<0, \quad DS=1; \\ >0 &{} \quad \text { otherwise}. \end{array} \right. \end{aligned}$$

Taxpayers are assumed to judge outcomes relative to a reference level of income *R*—the reference dependence element of PT. Thus, we write $\Delta Y^{i}\equiv Y^{i}-R$, $i=c,n$. We employ a specification of reference income that we shall show is sufficiently general to nest a wide range of those proposed in the existing literature. The underlying observation informing our specification is that existing approaches can be understood as weighted averages of wealth in the caught and not-caught states. Specifically, we write

2 $$\begin{aligned} R=\alpha \left( \cdot \right) Y^{c}+\left[ 1-\alpha \left( \cdot \right) \right] Y^{n}, \end{aligned}$$

where the function $\alpha \left( \cdot \right)$ satisfies

A5. $$\alpha \left( \cdot \right) =\phi _{1}+\frac{\phi _{2}}{ Y-X}$$,

and $\phi _{1}\left( \cdot \right)$ and $\phi _{2}\left( \cdot \right)$ are real-valued functions satisfying $\phi _{1X}=\phi _{2X}=0$ (where $\phi _{iX}\equiv \partial \phi _{i}\left( \cdot \right) /\partial X$). For future reference, we denote the elasticity of $\phi _{i}$ with respect to *t* as $\varepsilon _{\phi _{i},t}$. The specifications of reference income in the existing literature that are special cases of A5 are listed in Table 1. $R=0$, as under EUT, is the special case of A5 obtained by setting $\phi _{1}=t/\left[ ft+s\right]$ and $\phi _{2}=Y\left[ 1-t \right] /\left[ ft+s\right]$. Alternatively, setting $\phi _{1}=t/\left[ ft+s\right]$ and $\phi _{2}=0$, we obtain the taxpayer’s post-tax income if s/he does not evade (the legal post-tax income): $R=Y\left[ 1-t\right]$. This specification for reference income was first proposed by Dhami and al-Nowaihi (2007). It is also considered by Trotin (2012), and is one of the examples in the study of Hashimzade et al. (2013). Kőszegi and Rabin (2006) make a general argument that the reference level should reflect the expected outcome of the lottery. Consistent with such approaches, reference income is equated to the expected value of the gamble by setting $\phi _{1}=w\left( p\right)$ and $\phi _{2}=0$. Hashimzade et al. (2013) also consider the specification $R=X\left[ 1-t\right]$ as one of their examples, which obtains from A5 with $\phi _{1}=1/\left[ ft+s\right]$, and $\phi _{2}=0$. The specification of Yaniv (1999) exercises fully the generality of A5, for it implies $\phi _{2}\ne 0$. The as yet undefined notation in Table 1 required to characterise Yaniv’s specification we shall detail later.

**Table 1:** Characterising specifications of reference income in the literature

*R*	$\phi _{1}$	$\phi _{2}$
Constant ($=c$)	$\frac{t}{ft+s}$	$\frac{Y\left[ 1-t\right] -c}{ft+s}$
$Y\left[ 1-t\right]$	$\frac{t}{ft+s}$	0
$X\left[ 1-t\right]$	$\frac{1}{ft+s}$	0
Exp. val.	$w\left( p\right)$	0
Yaniv (1999)	$\frac{t}{ft+s}$	$\frac{t\left[ \omega b-Y\right] }{ft+s}$

Taxpayers are assumed to choose *X* to maximise

3 $$\begin{aligned} V=w\left( p\right) v\left( \Delta Y^{c}\right) +\left[ 1-w\left( p\right) \right] v\left( \Delta Y^{n}\right) , \end{aligned}$$

where, under A5,

$$\begin{aligned} \Delta Y^{n}=\left[ ft+s\right] \left[ \phi _{1}\left[ Y-X\right] +\phi _{2} \right] ; \quad \Delta Y^{c}=\left[ ft+s\right] \left[ \left[ \phi _{1}-1 \right] \left[ Y-X\right] +\phi _{2}\right] . \end{aligned}$$

Table 2 introduces four variants of the TBM, each of which may be considered with or without social stigma, and under either $p^{\prime }=0$ or $p^{\prime }<0$. These are only four of many possible variants, but are sufficient to make our key observations.

**Table 2:** Variants of the TBM

$\text {Variant}$	*R*	Diminishing sensitivity	Loss aversion	Probability weighting
EUT	0	No	No	No
$\mathrm{LA}\wedge \mathrm{PW}$	0	No	Yes	Yes
RD	$\in \left( Y^{c},Y^{n}\right)$	No	Yes	Yes
PT	$\in \left( Y^{c},Y^{n}\right)$	Yes	Yes	Yes

The simplest variant is EUT. To understand the marginal contribution of loss aversion and probability weighting, these features are introduced in the $\mathrm{LA}\wedge \mathrm{PW}$ variant. We introduce these together as it shall transpire that neither is important for the predictions of the TBM with respect to the Puzzle. Reference dependence is introduced $\text {in}$ variant RD, while the last variant, PT , satisfies the assumptions of cumulative prospect theory (Tversky and Kahneman 1992). As is widely noted, under diminishing sensitivity an interior maximum to (3) must satisfy $\Delta Y^{n}>0$ for $p^{{\prime }{\prime }}$ sufficiently small, for otherwise the taxpayer’s objective function is globally convex. Moreover, if $\Delta Y^{c}>0$, then the predictions of the PT variant are identical to those of the RD variant. Hence, under PT we assume $\Delta Y^{c}<0<\Delta Y^{n}$, and we retain this assumption in the RD variant to isolate the marginal effect of allowing for diminishing sensitivity. As the most parsimonious of the variants, EUT is at an inherent disadvantage relative to the remaining variants in respect of predicting empirical phenomena. This observation would make any failure of PT to outperform EUT the more surprising. As we shall now go on to demonstrate, these four variants of the TBM are consistent with special cases of A5.

Differentiating with respect to *X*, we obtain the first and second derivatives of (3) as

4 $$\begin{aligned} \frac{\partial V}{\partial X}= & {} \left[ ft+s\right] \left[ w\left( p\right) \left[ 1-\phi _{1}\right] v^{\prime }\left( \Delta Y^{c}\right) -\left[ 1-w\left( p\right) \right] \phi _{1}v^{\prime }\left( \Delta Y^{n}\right) \right] \nonumber \\&-\;p^{\prime }w^{\prime }\left[ v\left( \Delta Y^{n}\right) -v\left( \Delta Y^{c}\right) \right] ; \end{aligned}$$

5 $$\begin{aligned} \frac{\partial ^{2}V}{\left[ \partial X\right] ^{2}}\equiv & {} D=\left[ ft+s \right] ^{2}\left[ w\left( p\right) \left[ 1-\phi _{1}\right] ^{2}v^{\prime \prime }\left( \Delta Y^{c}\right) +\left[ 1-w\left( p\right) \right] \left[ \phi _{1}\right] ^{2}v^{\prime \prime }\left( \Delta Y^{n}\right) \right] \nonumber \\&+\;2\left[ ft+s\right] p^{\prime }w^{\prime }\left[ \left[ 1-\phi _{1}\right] v^{\prime }\left( \Delta Y^{c}\right) +\phi _{1}v^{\prime }\left( \Delta Y^{n}\right) \right] \nonumber \\&-\;\left[ p^{\prime \prime }w^{\prime }+\left[ p^{\prime }\right] ^{2}w^{\prime \prime }\right] \left[ v\left( \Delta Y^{n}\right) -v\left( \Delta Y^{c}\right) \right] . \end{aligned}$$

Sufficient conditions for a (global) interior maximum with respect to Eqs. (4) and (5) are $\partial V/\partial X=0$ and the second derivative $D<0$ for all *X*. Under diminishing sensitivity, the second-order condition ($D<0$)—implying that the objective function is concave in *X*—cannot be guaranteed for any easily interpretable restriction on the parameters. Moreover, under diminishing sensitivity it is possible—because of the possibility of corner solutions—that the first- and second-order conditions do not describe the solution of the maximisation problem.^8^ As these difficulties of PT are well understood, we choose to set them aside here. Henceforth, when analysing the TBM under diminishing sensitivity, we proceed under the maintained assumption that the second derivative in (5) is negative, such that indeed the first-order condition describes a unique choice for the taxpayer on the interval $X\in \left( 0,Y\right)$.^9^

A6. $$D<0$$ for all *X*.

If $\phi _{1}$ is decreasing in stigma ($\phi _{1s}<0$), as is the case for all the specifications of reference income in Table 1, then the restriction to interior solutions for evasion places an upper bound $s<{\overline{s}}$ on the level of stigma. We prove the following Lemma, which extends Dhami and al-Nowaihi’s Proposition 5:

### Lemma 1

If $\phi _{1s}<0$ then, at any interior maximum of (3), it must hold that $s<{\overline{s}}$, where ${\overline{s}}$ is the unique *s* such that

$$\begin{aligned} \phi _{1}\left( {\overline{s}}\right) =\left\{ \begin{array}{ll} \lim _{X\uparrow Y}\frac{\lambda w\left( p\left( X\right) \right) }{1+\left[ \lambda -1\right] w\left( p\left( X\right) \right) } &{} \quad \text { if } \; \phi _{2}=0; \\ \lim _{X\uparrow Y}w\left( p\left( X\right) \right) &{} \quad \text { otherwise}. \end{array} \right. \end{aligned}$$

## Analysis

### Exogenous audit probability

In this section, we examine the four variants of the TBM given in Table 2 under one further assumption regarding the probability of audit:

A7. $$p^{\prime }=0$$,

which is equivalent to the assumption of random auditing. As we restrict our attention to interior maxima, and several variants in Table 2 additionally require $\Delta Y^{c}<0<\Delta Y^{n}$, we establish the conditions under which each of these restrictions is satisfied:

#### Lemma 2

Under A0–A7,

(i) at any interior maximum of (3), it must hold that $\phi _{1}\in \left( 0,1\right)$;
(ii) $\Delta Y^{c}<0<\Delta Y^{n}$ always holds at an interior maximum of (3) if and only if $\phi _{2}=0$.

Part (ii) of Lemma 2 weakens the conditions for an interior maximum to exist given in Proposition 3 of Dhami and al-Nowaihi (2007).

#### Proposition 1

If assumptions A0–A7 hold then, at an interior maximum,

(i) under both EUT and ${LA}\wedge {PW}$, $\frac{\partial X}{\partial t} \ge 0$ for *s* sufficiently close to (or equal to) zero and $\frac{\partial X}{\partial t}<0$ for *s* sufficiently close to ${\overline{s}}$;
(ii) under RD there exists a $\Phi _{\left( \mathrm{ii}\right) }>0$ such that $\frac{\partial X}{\partial t}\gtreqless 0\Leftrightarrow \phi _{1t}\lesseqgtr \Phi _{\left( \mathrm{ii}\right) }$;
(iii) under PT there exists a critical value of $\phi _{1t}$, $\Phi _{\left( \mathrm{iii}\right) }$, which may be positive or negative, around which $\frac{\partial X}{\partial t}$ switches sign. For *s* sufficiently close to ${\overline{s}}$ (or for $\phi _{1}$ sufficiently close to zero), $\frac{\partial X}{\partial t}\gtreqless 0\Leftrightarrow \phi _{1t}\lesseqgtr \Phi _{\left( \mathrm{iii}\right) }>\Phi _{\left( \mathrm{ii}\right) }$;
(iv) under both PT and RD, $\frac{\partial X}{\partial t} \ge 0$ for $\phi _{1t}$ sufficiently close to (or equal to) zero;
(v) under Yaniv’s (1999) specification of reference income there exists a $\Phi _{\left( v\right) }>-\frac{tf}{tf+s}$ such that $\frac{\partial X}{\partial t }\gtreqless 0\Leftrightarrow \varepsilon _{\phi _{2},t}\lesseqgtr \Phi _{\left( v\right) }$.

Part (i) of Proposition 1 begins by extending Yitzhaki’s (1974) statement of the Puzzle—which implicitly assumes $\lambda =1$, $s=0$ and $w\left( p\right) =p$—to allow for loss aversion, probability weighting and for sufficiently low levels of stigma. The finding in part (i) is a pure income effect: there is no substitution effect as both the tax rate and the penalty rate increase proportionally with *t*. The income effect generated by an increase in *t* can itself be considered in two parts. The first part, $IE_{\phi }$, is the part of the income effect arising from movements in *R* through $\left\{ \phi _{1},\phi _{2}\right\}$; and the second part, $IE_{-\phi }$, is the remainder of the effect—arising directly from movements in *t* and from the equilibrium adjustments of *X*. $IE_{-\phi }$ always increases the optimal declaration following a tax rate rise at an interior maximum, whereas $IE_{\phi }$ under both EUT and $\mathrm{LA}\wedge \mathrm{PW}$ is zero; hence, the Yitzhaki puzzle.

There are two elements of PT that can reverse the above intuition. Reference dependence can reverse the Puzzle if it makes taxpayers feel richer in expectation, rather than poorer, following an increase in the tax rate. To make these concepts precise, we define the expected absolute wealth of a taxpayer as ${\mathbf {E}}\left( Y\right) \equiv w\left( p\right) Y^{c}+ \left[ 1-w\left( p\right) \right] Y^{n}$ and the expected relative wealth (when wealth is measured relative to the reference level *R*) as ${\mathbf {E}} \left( \Delta Y\right) \equiv w\left( p\right) \Delta Y^{c}+\left[ 1-w\left( p\right) \right] \Delta Y^{n}$. We write that taxpayers “feel richer in expectation” when ${\mathbf {E}}\left( \Delta Y\right)$ increases.

Taxpayers can be expected to be left poorer in absolute terms from a tax rate rise, even after accounting for equilibrium adjustments in *X*. The only possible exception is the (seemingly unlikely) case in which the government operates at a tax rate on the “wrong” side of a Laffer curve relationship for expected revenue (whereby $$\partial X/\partial t$$ is sufficiently negative that also $$\partial \left[ tX\right] /\partial t<0$$, such that taxpayers pay less tax following a tax rate rise).^10^ Under EUT we have $$ R=0$$ so when taxpayers are made poorer in absolute terms from a tax rate rise, they are also poorer in relative terms. Yet, under reference dependence, it is possible for an increase in the tax rate to make taxpayers feel *richer* in relative terms, even when they are made poorer in absolute terms, if reference income falls faster with the tax rate than does post-tax income. When taxpayers feel richer following a tax rate rise, the income effect drives declared income down, rather than up, creating an opening to resolve the Puzzle (the RD-effect). Under what conditions will an increase in the tax rate result in expected relative income, $${\mathbf {E}}\left( \Delta Y\right) $$, (and relative income in the not-caught state, $$\Delta Y^{n}$$) increasing too? We now show that the answer to this question is regulated by the sign of $$\phi _{1t}$$. In the following Lemma, we write “*d*” rather than the partial derivative symbol “$$\partial $$ ” to emphasise that here we consider the “full” effect of a change in *t*, including that arising from any equilibrium adjustment in *X*.

#### Lemma 3

Under the assumptions of Proposition 1, it holds that

(i) Under RD $$\frac{\mathrm{d}\left[ {\mathbf {E}}\left( \Delta Y\right) \right] }{\mathrm{d}t}\gtreqless 0\Leftrightarrow \frac{\mathrm{d}\left[ \Delta Y^{n}\right] }{\mathrm{d}t}\gtreqless 0\Leftrightarrow \phi _{1t}\gtreqless 0$$;
(ii) Under PT the sign of $\frac{\mathrm{d}\left[ {\mathbf {E}}\left( \Delta Y\right) \right] }{\mathrm{d}t}$ and $\frac{\mathrm{d}\left[ \Delta Y^{n}\right] }{\mathrm{d}t}$ switch around $\phi _{1t}=0$. For *s* sufficiently close to ${\overline{s}}$, $\frac{\mathrm{d}\left[ {\mathbf {E}}\left( \Delta Y\right) \right] }{\mathrm{d}t}\gtreqless 0\Leftrightarrow \frac{\mathrm{d}\left[ \Delta Y^{n}\right] }{\mathrm{d}t}\gtreqless 0\Leftrightarrow \phi _{1t}\gtreqless 0$.

With Lemma 3 in hand, it is straightforward to interpret part (ii) of Proposition 1, which states that a necessary condition for the RD variant to reverse the Puzzle is that $\phi _{1t}>0$. By part (i) of Lemma 3, this condition is equivalent to the statement that a tax rate rise makes taxpayers feel richer in expectation. As a sufficient condition to reverse the Puzzle, $\phi _{1t}$ must not just be positive, but sufficiently so to overcome an offsetting substitution effect. How plausible is the necessary assumption that taxpayers feel richer in the not-caught state, and in expectation, following a tax rate rise? Barring the Laffer curve consideration given above, the taxpayer—judged in absolute terms—is poorer in expectation following a tax rate increase, in which case the RD-effect relies on a strong disjunction between the response to a tax rate rise of absolute and relative income to resolve the Yitzhaki puzzle. Although we know of no direct empirical evidence on this point, we find the idea that taxpayers made poorer in absolute terms would also feel poorer (i.e., $\mathrm{d}\left[ \mathbf { E}\left( Y\right) \right] /\mathrm{d}t<0\Rightarrow \mathrm{d}\left[ {\mathbf {E}}\left( \Delta Y\right) \right] /\mathrm{d}t<0$) compelling from a psychological perspective.

The second element of PT that can reverse the Yitzhaki puzzle is diminishing sensitivity: this can overturn the Puzzle by reversing the sign of the income effect in the caught state, so that when a taxpayer feels poorer in the caught state, they are induced to take more (not less) risk, and thus declare less income (the DS-effect). Note, however, that whereas the RD-effect requires that a tax rate rise makes taxpayers feel richer, the DS-effect requires that a tax rate rise makes taxpayers feel poorer. Equivalently, reference dependence requires reference income to be sensitive to the tax rate to resolve the Puzzle, but diminishing sensitivity requires reference income to be insensitive to the tax rate. Therefore, the effects of reference dependence and diminishing sensitivity with respect to the Puzzle oppose each other. Accordingly, part (ii) of Lemma 3 implies that an increase in $\phi _{1t}$ (which makes reference income more sensitive to the tax rate) can either increase or decrease the effect of a tax rate rise on expected income depending on the balance of the DS- and RD-effects. It is this ambiguity that lies at the heart of part (iii) of Proposition 1, which gives the condition needed for the DS-effect to reverse the Puzzle.

Part (iv) of the Proposition exploits the observation that the RD- and DS-effects are in opposition to establish a simple and relevant condition under which the Yitzhaki puzzle unambiguously holds. It states that when the expected value of the gamble moves sufficiently little with the tax rate ($\phi _{1t}$ close to zero), the Yitzhaki puzzle holds under both the PT and RD variants. In this case, taxpayers neither feel sufficiently richer after a tax rate rise for the RD-effect to overturn the Puzzle, nor feel sufficiently poorer for the DS-effect to overturn the Puzzle.

How does introducing stigma mediate the above findings? Part (i) of Proposition 1 states that the Yitzhaki puzzle is always resolved under EUT for *s* sufficiently high. This finding is consistent with various analyses incorporating stigma—differing in the way in which stigma enters the taxpayer’s objective function—that establish bounds for *s* above which $\partial X/\partial t<0$ (al-Nowaihi and Pyle 2000; Dell’Anno 2009; Gordon 1989; Kim 2003). In contrast, even in the neighbourhood of the maximum level of stigma, the Puzzle is not unambiguously reversed in either the PT or RD variants. Rather, close to the maximum level of stigma, part (iii) of Proposition 1 states that the same questionable necessary condition for reversing the Yitzhaki puzzle under the RD variant holds also under PT: taxpayers must feel richer in the not-caught state, and in expectation, after an increase in the tax rate (and, as a sufficient condition, this effect must be sufficiently strong).

#### Implications for the literature

We now consider the implications of our findings for the existing literature. We first consider those specifications of reference income given in Table 1 that satisfy $R\in \left( Y^{c},Y^{n}\right)$. To begin, we consider these specifications in the absence of stigma ($s=0$). In this case, the specification of reference income as the taxpayer’s legal post-tax income, $R=Y\left[ 1-t\right]$, implies $\phi _{1}=f^{-1}$, and the specification as the expected value of the tax gamble implies $\phi _{1}=w\left( p\right)$. It is readily observed that, for both specifications, $\phi _{1t}=0$, thus (by part (iv) of Proposition 1) the Yitzhaki puzzle holds both under the PT and RD variants.^11^ Only for the specification of reference income $R=X\left[ 1-t\right]$ examined by Hashimzade et al. (2013) does part (iv) of Proposition 1 not apply, for it implies $\phi _{1}=\left[ ft\right] ^{-1}$, hence $\phi _{1t}=-\left[ ft^{2}\right] ^{-1}<0$. In this case, the Yitzhaki puzzle holds under RD, but the sign of $\partial X/\partial t$ is not determined a priori under PT. Thus, for two of the three specifications of reference income in the literature satisfying $R\in \left( Y^{c},Y^{n}\right)$—including the most utilised specification of reference income as the taxpayer’s legal post-tax income—neither PT nor its stripped-down variants resolve the Yitzhaki puzzle. Note that, for these two specifications that do not resolve the Puzzle, the ability of the PT and RD variants to reverse the Yitzhaki puzzle is strictly weaker than that of EUT. The latter can always reverse the Puzzle, albeit by invoking the empirically unsatisfactory assumption of increasing absolute risk aversion (and this must be sufficiently strong), whereas PT and its variants cannot reverse the Puzzle for any choice of preferences consistent with an interior maximum.

Allowing for stigma yields mixed findings. For the specification of reference income as legal post-tax income, the predictions of the PT and RD variants are improved, for we obtain $\phi _{1t}=s/\left[ ft+s\right] ^{2}>0$, such that the sign of $\partial X/\partial t$ becomes ambiguous in general, but unambiguously negative for *s* close to $\overline{ s}$. This reversal as $s\uparrow {\overline{s}}$ relies, however, on a tax rate rise making taxpayers feel richer ($\phi _{1t}>0$). The predictions for $\partial X/\partial t$ are unchanged for the specification of reference income as the expected value of the gamble, however, and for the specification $R=X\left[ 1-t\right]$ they worsen: in the absence of stigma this specification implies an ambiguous sign for $\partial X/\partial t$ under PT, but unambiguously implies Yitzhaki’s puzzle for *s* close to ${\overline{s}}$. In summary, augmenting EUT with stigma unambiguously improves its descriptive abilities, for it always overturns the Yitzhaki puzzle if enough stigma is allowed for. Augmenting PT with stigma sharpens its predictions with respect to the Yitzhaki puzzle, but does not unambiguously improve their descriptive validity. In particular, for either the PT or RD variants to overturn the Puzzle, even close to the maximum level of stigma, an increase in the tax rate must make taxpayers feel richer in expectation.

Proposition 1 connects to two further studies in the existing literature: Yaniv (1999) and Bernasconi and Zanardi (2004), each of which employs a specification of reference income that does not always satisfy $R\in \left( Y^{c},Y^{n}\right)$. Specifically, Yaniv examines a model with reference income specified as $R=Y-H$, where *H* is the amount of an advance tax payment. The advance payment *H* is specified (up to a constant) as $H=\omega tb$ where *b* is the tax authority’s estimate of the taxpayer’s income (which could under- or over-estimate the true *Y*), and $\omega \in \left[ 0,1\right]$. This specification of reference income is a special case of A5 with $\phi _{1}=t/\left[ tf+s\right]$ and $\phi _{2}=t\left[ \omega b-Y\right] /\left[ ft+s\right]$. Yaniv’s model is not, however, encompassed by the TBM for, in forming the taxpayer’s objective function, the author adopts the segregation assumption of the original 1979 version of PT, according to which certain gains and losses are extracted from the gamble. The coding phase (in which the original version of PT held segregation to occur) is, however, de-emphasised in the later cumulative version of PT—the version we employ. Accordingly, the remaining literature on PT and tax evasion, all of which post-dates Yaniv’s contribution, does not adopt this assumption. Does Yaniv’s model still reverse the Yitzhaki puzzle if segregation is not assumed? In part (v) of Proposition 1, we give a result that addresses this question, and answers it in the negative: for Yaniv’s specification of *R* we obtain $\varepsilon _{\phi _{2},t}=-1$ yet, by part (v), a necessary (and still not sufficient) condition for the Yitzhaki puzzle to be overturned is $\varepsilon _{\phi _{2},t}>-tf/\left[ tf+s\right] \ge -1$.

The last contribution to the literature we discuss here is that of Bernasconi and Zanardi (2004). Unlike the rest of the literature, this study does not explicitly specify reference income, but rather it examines taxpayer behaviour for all possible values of *R*. Implicitly, however, the non-specification of *R* is equivalent to the specification of *R* as a constant, $R=c$. Viewed this way, the Bernasconi and Zanardi specification of *R* is the special case of A5 with $\phi _{1}=t/\left[ ft+s\right]$ and $\phi _{2}=\left[ Y\left[ 1-t\right] -c\right] /\left[ ft+s\right]$. As this specification of reference income implies $R_{t}=0$ the RD-effect does not arise, leaving only a pure DS-effect. Hence, under the PT variant, $\partial X/\partial t<0$. The contribution of Proposition 1 in respect of this analysis is to highlight that, once an endogenous specification for *R* is adopted, PT and its variants may no longer reverse the Yitzhaki puzzle. As the authors note in their conclusion, pinning-down reference income is unavoidable if PT is to yield clear and testable predictions.

We complete our discussion of Proposition 1 by assessing its implications for the separate elements of the TBM. The proposition makes clear that the Yitzhaki puzzle can be resolved without recourse to either probability weighting or loss aversion, and part (i) makes clear that when EUT is augmented with probability weighting and/or loss aversion the Puzzle is unaffected. These two features of PT are, therefore, unimportant with respect to the predicted sign of $\partial X/\partial t$.^12^ Diminishing sensitivity, too, is neither necessary nor sufficient to resolve the Puzzle. Indeed, in the presence of social stigma, diminishing sensitivity appears to hinder the resolution of the Puzzle. To see this, note from part (iv) of Proposition 1 that, close to the maximum level of social stigma, the necessary condition for the Puzzle to be resolved is weaker under RD than under PT. Thus, reference dependence stands out as the only feature of PT that is essential to resolving the Puzzle.

### Endogenous audit probability

In practice only a fairly small proportion of tax authority audits are selected randomly. Accordingly, here we repeat the analysis conducted in the previous section under an alternative assumption to that of A7:

A8. $$p^{\prime }<0$$.

Under A8, Dhami and al-Nowaihi (2007) prove (their Proposition 1c) that if the only assumptions made with regard to preferences are A0, A2 and A3, then the sign of $\partial X/\partial t$ under EUT is ambiguous. To obtain comparative static results under A8, we require a stronger restriction on preferences. Homogeneous utility appears of particular relevance in this context as Tversky and Kahneman’s (1992) original value function—subsequently axiomatised under PT by al-Nowaihi et al. (2008)—is piecewise homogeneous. Accordingly, we assume

A9. $$xu^{\prime }\left( x\right) =\left\{ \begin{array}{cl} \beta u\left( x\right) &{} \quad \text { if }\; x\ge 0; \\ \gamma u\left( x\right) &{} \quad \text { otherwise}; \end{array} \right. $$

which is equivalent to the statement that $u\left( x\right)$ is homogeneous of degree $\beta$ in the gain domain, and of degree $\gamma$ in the loss domain.^13^ For A9 to be compatible with A2, we require $\beta \in \left( 0,1\right)$ and $\gamma >1$.

Whereas, when $p^{\prime }=0$, $\phi _{1}\in \left( 0,1\right)$ was guaranteed by Lemma 2, under A8, interior maxima satisfying $\phi _{1}>1$ can exist. This is readily observed from the first-order condition in equation (4), where the final term is positive when $p^{\prime }<0$. We now show, however, that under the stronger preference restriction in A9 $\phi _{1}\in \left( 0,1\right)$ must hold at an interior maximum when $\phi _{2}=0$.

#### Lemma 4

Under A0–A6 and A8–A9, and assuming $\phi _{2}=0$, at any interior maximum of (3), it must hold that $\phi _{1}\in \left( 0,1\right)$.

Given Lemma 4, it is straightforward to verify that $\phi _{2}=0$ remains necessary and sufficient for $\Delta Y^{c}<0<\Delta Y^{n}$ to hold at any interior maximum.

#### Proposition 2

If assumptions A0–A6 and A8–A9 hold then, at an interior maximum,

(i) under both EUT and LA$\wedge$PW $\frac{\partial X}{ \partial t}\ge 0$ for *s* sufficiently close to (or equal to) zero, and $\frac{\partial X}{\partial t}<0$ for *s* sufficiently close to ${\overline{s}}$;
(ii) under PT $\frac{\partial X}{\partial t}\gtreqless 0\Leftrightarrow \phi _{1t}\lesseqgtr 0$;
(iii) under RD $\frac{\partial X}{\partial t}\gtreqless 0\Leftrightarrow \phi _{1t}\lesseqgtr \frac{f\left[ \gamma -\beta \right] \phi _{1}\left[ \phi _{1}-1\right] }{\left[ tf+s\right] \left[ \gamma \phi _{1}-\beta \left[ \phi _{1}-1\right] \right] }<0$.

Part (i) of Proposition 2 extends the original statement of the Yitzhaki puzzle to the case of variable audit probability and homogeneous utility (as well as allowing for loss aversion, probability weighting, and sufficiently low levels of stigma). Introducing variable audit probabilities does not, therefore, appear to enhance importantly the descriptive abilities of EUT with respect to the Puzzle. Under PT, the income and substitution effects cancel, leaving a residual that takes the sign of $-\phi _{1t}$. A simple necessary and sufficient condition for the Yitzhaki puzzle to be resolved, therefore, holds: $\phi _{1t}>0$. In the absence of stigma, all three of the specifications of reference income satisfying $R\in \left( Y^{c},Y^{n}\right)$ in the existing literature fail to satisfy this necessary condition. Moreover, it is straightforward to show (with the same procedure we use to prove Lemma 3) that when $\phi _{1t}>0$ it holds that both $\mathrm{d}\left[ {\mathbf {E}}\left( \Delta Y\right) \right] /\mathrm{d}t$ and $\mathrm{d}\left[ \Delta Y^{n}\right] /\mathrm{d}t$ are positive, so whenever the Puzzle is resolved under PT it also predicts that expected relative wealth increases following a tax rate rise. Thus, allowing for variable audit probabilities does not appear to enhance the descriptive abilities of PT and its variants with respect to the Puzzle either. Once again, the condition for the RD variant to overturn the Yitzhaki puzzle is strictly weaker than that for PT.

The effects of stigma are similar to those in the analysis with fixed probabilities. In particular, in the presence of stigma, the specification of reference income as the legal post-tax income satisfies the necessary condition for Yitzhaki’s puzzle to be resolved, but the predictions regarding $\partial X/\partial t$ of the PT and RD variants for the remaining two specifications of reference income satisfying $R\in \left( Y^{c},Y^{n}\right)$ are either unchanged with stigma, or worsen with stigma.

What are the implications of Proposition 2 for the existing literature? The model of Dhami and al-Nowaihi (2007) satisfies the assumptions of Proposition 2, where it corresponds to a special case of the PT variant with $R=Y\left[ 1-t\right]$. Our insights with respect to this contribution are twofold. First, while these authors demonstrate, in this special case, that PT outperforms EUT, we can assess whether PT has a general advantage over EUT in relation to predicting the sign of $\partial X/\partial t$. Concerning this question, Proposition 2 does not suggest that PT has such an advantage.^14^ Second, the specification of the model implies that taxpayers who are made poorer in absolute terms by a tax rate rise will feel relatively richer.

## Conclusion

Prospect theory (PT) is widely viewed as the best available description of how (many) people behave in risky settings, and we do not dissent from this view. Barberis (2013) notes, however, that PT is not always straightforward to apply: in particular, the most appropriate specification of the reference level is often unclear.^15^ Yet, we show that theoretical predictions related to tax evasion and the Yitzhaki puzzle depend crucially on the specification of that reference level.

We focus on tax evasion and in particular on the Yitzhaki puzzle: the EUT model of tax evasion predicts a decrease in tax evasion when the tax rate increases ($\partial X/\partial t<0$). The PT literature provides several specific examples in which the Puzzle is solved: our research is a first step towards understanding, in a general setting, the conditions under which PT reverses the Puzzle. Furthermore, by stripping down the elements of PT, we are able to (i) identify the forces that drive predictions concerning the impact of the tax rate on tax evasion; (ii) compare our findings with those under Expected Utility Theory (EUT). Although our findings do not clearly endorse the descriptive validity of EUT, we find that, in respect of the Puzzle, many specifications of PT share similar descriptive deficiencies. Interestingly, the set of specifications of PT that cannot reverse the Puzzle overlaps with many of those advocated in the literature. The auxiliary assumptions we consider—social stigma and variable audit probability—allow PT in some cases to reverse the Yitzhaki puzzle, but when they do so, they also allow the model under EUT to do likewise when similarly augmented. Although there are indeed many specifications of PT that do reverse the Puzzle, we find that in many instances these specifications imply that taxpayers will feel richer after a tax rise, for they imply that reference income falls (and by a sufficient amount) following a tax rate increase. That taxpayers made objectively poorer by a tax rate rise would feel subjectively richer seems psychologically questionable, albeit empirical work addressing this point is needed. Thus, Barberis’s point concerning the difficulty of properly identifying the reference level is evident in the tax evasion context.

Our findings contribute to the literature on the individual elements of PT. Of the four elements of PT that we isolate, only reference dependence is necessary to resolve the Puzzle, which is in agreement with its supposed status as the most accepted of all the elements of PT. Loss aversion and probability weighting are largely irrelevant for the sign of $\partial X/\partial t$. Invoking Occam’s razor, we believe that some of the findings relating to the Yitzhaki puzzle that have been attributed to PT may more properly be interpreted as being attributable to simpler reference-dependent models that contain only a subset of the elements of PT. Perhaps more surprisingly, diminishing sensitivity is neither necessary nor sufficient to resolve the Yitzhaki puzzle, and indeed pushes $\partial X/\partial t$ the “wrong” way in the presence of social stigma.

Given the importance of PT in explaining behaviour in so many economic domains, it seems altogether likely that it is also of importance in explaining behaviour towards tax evasion too. As such, our cautionary findings need not imply that tax evasion researchers should cease to explore the insights of PT, but they do suggest that renewed focus must be placed on the specification of reference income and preferences. In particular, if researchers wish to retain the common assumption of homogeneous preferences then specifications of reference income beyond those encompassed by our model must be sought, for otherwise taxpayers must feel subjectively richer after a tax rate rise for PT to reverse the Puzzle.

## Appendix

### Proof of Lemma 1

Under A6 the first derivative in (4) is decreasing, and when an interior maximum exists it must also switch sign on $\left( 0,Y\right)$. Hence, $\lim _{X\downarrow 0}\partial V/\partial X\ge 0$ and $\lim _{X\uparrow Y}\partial V/\partial X\le 0$. Taking the limit of (4) as $X\uparrow Y$, and noting that $\lim _{X\uparrow Y}\left\{ v\left( \Delta Y^{n}\right) -v\left( \Delta Y^{c}\right) \right\} =0$ by A1, the latter inequality in the preceding sentence implies

$$\begin{aligned} \phi _{1}\ge \lim _{X\uparrow Y}\frac{w\left( p\left( Y\right) \right) v^{\prime }\left( \Delta Y^{c}\right) }{\left[ 1-w\left( p\left( Y\right) \right) \right] v^{\prime }\left( \Delta Y^{n}\right) +w\left( p\left( Y\right) \right) v^{\prime }\left( \Delta Y^{c}\right) }. \end{aligned}$$

Using (1), this may be re-written as

$$\begin{aligned} \phi _{1}\ge \left\{ \begin{array}{ll} \lim _{X\uparrow Y}\frac{\lambda w\left( p\left( X\right) \right) }{1-w\left( p\left( X\right) \right) +\lambda w\left( p\left( X\right) \right) } &{} \quad \text { if }\;\phi _{2}=0; \\ \lim _{X\uparrow Y}w\left( p\left( X\right) \right) &{} \quad \text { otherwise.} \end{array} \right. \end{aligned}$$

$$\square $$

### Proof of Lemma 2

(i) Suppose, by way of contradiction, $\phi _{1}\ge 1$ then the first derivative in (4) with $p^{\prime }=0$ is always negative, thus ruling out the possibility of an interior maximum. Hence, $\phi _{1}<1$ at an interior maximum. Next, the proof of Lemma 1 establishes that either $\phi _{1}\ge \lim _{X\uparrow Y}w\left( p\left( X\right) \right) >0$ or

$$\begin{aligned} \phi _{1}\ge \lim _{X\uparrow Y}\frac{\lambda w\left( p\left( X\right) \right) }{1-w\left( p\left( X\right) \right) +\lambda w\left( p\left( X\right) \right) }\ge w\left( p\left( X\right) \right) >0. \end{aligned}$$

Hence, $$\phi _{1}>0$$.

(ii) We first show that $\phi _{2}=0$ implies that $Y^{c}<R<Y^{n}$. Equation (2) becomes $R=\phi _{1}Y^{c}+\left[ 1-\phi _{1}\right] Y^{n}$. By Lemma 2, $\phi _{1}\in \left( 0,1\right)$, hence *R* is a convex combination of $Y^{n}$ and $\ Y^{c}$.

We now show that $Y^{c}<R<Y^{n}$ for all $X\in \left( 0,Y\right)$ implies $\phi _{2}=0$. $\Delta Y^{n}>0>\Delta Y^{c}$ holds for all $X\in \left( 0,Y\right)$. At $X=Y$, however, it holds that $Y^{n}=Y^{c}=Y\left[ 1-t \right]$, so $\Delta Y^{n}=\Delta Y^{c}=0$. Therefore, as $R\in \left[ Y^{n},Y^{c}\right]$ arbitrarily close to $X=Y$, by continuity, we must have $R=Y^{n}=Y^{c}=Y\left[ 1-t\right]$ at $X=Y$. However, when $X=Y$, Eq. (2) gives $R=Y\left[ 1-t\right] -\left[ ft+s\right] \phi _{2}$, which is consistent with $R=Y\left[ 1-t\right]$ also holding only if $\left[ ft+s\right] \phi _{2}=0$, hence $\phi _{2}=0$. $\square$

### Proof of Proposition 1

We start by proving statement (i). Under the parameter restrictions defining $\mathrm{LA}\wedge \mathrm{PW}$ the derivative $\partial X/\partial t$, found implicitly from (4), is

$$\begin{aligned} \frac{\partial X}{\partial t}= & {} -\frac{1}{D}\left[ \left[ 1\!-\!w\left( p\right) \right] tXv^{\prime \prime }\left( Y^{n}\right) \!-\!w\left( p\right) \left\{ t\left[ f-1 \right] +s\right\} \left[ X\!+\!f\left[ Y-X\right] \right] v^{\prime \prime }\left( Y^{c}\right) \right. \nonumber \\&\left. +\;w\left( p\right) \left[ f-1\right] v^{\prime }\left( Y^{c}\right) -\left[ 1-w\left( p\right) \right] v^{\prime }\left( Y^{n}\right) \right] \text {.} \end{aligned}$$

Substituting from the first-order condition, we obtain

$$\begin{aligned} \frac{\partial X}{\partial t}= & {} -\frac{1}{D}\left\{ \phantom {\frac{w\left( p\right) s}{t}v^{\prime }\left( Y^{c}\right) }\left[ 1-w\left( p\right) \right] tv^{\prime }\left( Y^{n}\right) \left[ Y\left[ A\left( Y^{c}\right) -A\left( Y^{n}\right) \right] +\left[ Y-X\right] \left[ A\left( Y^{n}\right) +\left[ f-1\right] A\left( Y^{c}\right) \right] \right] \right. \nonumber \\&\left. -\frac{w\left( p\right) s}{t}v^{\prime }\left( Y^{c}\right) \right\} . \end{aligned}$$

If $s=0$ this rewrites as

A.1 $$\begin{aligned} \frac{\partial X}{\partial t}=\frac{1}{t}\left[ \left[ Y-X\right] +\frac{Y \left[ A\left( Y^{c}\right) -A\left( Y^{n}\right) \right] }{\left[ f-1\right] A\left( Y^{c}\right) +A\left( Y^{n}\right) }\right] . \end{aligned}$$

Under A4, $A\left( Y^{c}\right) -A\left( Y^{n}\right) \ge 0$, hence $\partial X/\partial t>0$. As equation (A.1) holds for $w\left( p\right) =p$, and is independent of $\lambda$, $\partial X/\partial t\ge 0$ also holds under EUT. If $s\uparrow {\bar{s}}$ then $X\uparrow Y$ and $A\left( Y^{c}\right) \downarrow A\left( Y^{n}\right)$ so

$$\begin{aligned} \frac{\partial X}{\partial t}=\lim _{s\uparrow {\bar{s}}}\frac{w\left( p\right) s}{tD}v^{\prime }\left( Y^{c}\right) <0. \end{aligned}$$

(ii–iv). Under the relevant parameter restrictions the derivative $\partial X/\partial t$, found implicitly from the first-order condition, is

$$\begin{aligned}&\frac{\partial X}{\partial t} = \frac{\left[ ft+s\right] }{D} \\&\quad \times \left[ \begin{array}{c} \phi _{1t}\left[ w\left( p\right) v^{\prime }\left( \Delta Y^{c}\right) + \left[ 1-w\left( p\right) \right] v^{\prime }\left( \Delta Y^{n}\right) \right] \\ +\left[ Y-X\right] \left[ \begin{array}{c} w\left( p\right) \left[ \phi _{1}-1\right] \left[ f\left[ \phi _{1}-1\right] +\phi _{1t}\left[ ft+s\right] \right] v^{\prime \prime }\left( \Delta Y^{c}\right) \\ +\phi _{1}\left[ 1-w\left( p\right) \right] \left[ f\phi _{1}+\phi _{1t} \left[ ft+s\right] \right] v^{\prime \prime }\left( \Delta Y^{n}\right) \end{array} \right] \end{array} \right] . \end{aligned}$$

After some algebra and using the first-order condition to simplify the equation, we obtain:

A.2 $$\begin{aligned} \frac{\partial X}{\partial t}= & {} \frac{f\left[ Y-X\right] }{ft+s}+\frac{ \left[ ft+s\right] \phi _{1t}}{D}\left[ \frac{1}{\phi _{1}}\left[ w\left( p\right) v^{\prime }\left( \Delta Y^{c}\right) \right] \right. \nonumber \\&\left. +\;\phi _{1}\left[ ft+s\right] \left[ Y-X\right] \left[ 1-w\left( p\right) \right] v^{\prime }\left( \Delta Y^{n}\right) \left[ A\left( \Delta Y^{c}\right) -A\left( \Delta Y^{n}\right) \right] \right] .\nonumber \\ \end{aligned}$$

Equation (A.2) allows us to prove parts (ii–iv). Notice that the first term in (A.2) is always positive. The second term is all multiplied by $\phi _{1t}$. This is sufficient to prove statement (iv). Indeed, when $\phi _{1t}=0$, then the second term disappears and $\partial X/\partial t\ge 0$. By continuity, this result must also hold in a neighbourhood of 0.

Under RD, $A\left( \Delta Y^{c}\right) -A\left( \Delta Y^{n}\right) \ge 0$, so the sign of the second term in (A.2) is that of $-\phi _{1t}$. Define $\Phi _{\left( \mathrm{ii}\right) }$ as the value of $\phi _{1t}$ such that $\partial X/\partial t=0$. Then,

$$\begin{aligned} \Phi _{\left( \mathrm{ii}\right) }= & {} -\frac{f\left[ Y-X\right] D}{\left[ ft+s\right] ^{2}} \\&\times \;\left[ \frac{w\left( p\right) v^{\prime }\left( \Delta Y^{c}\right) }{ \phi _{1}}+\phi _{1}\left[ ft+s\right] \left[ Y-X\right] \left[ 1-w\left( p\right) \right] v^{\prime }\left( \Delta Y^{n}\right) \left[ A\left( \Delta Y^{c}\right) -A\left( \Delta Y^{n}\right) \right] \right] ^{-1}. \end{aligned}$$

Notice that $\Phi _{\left( \mathrm{ii}\right) }>0$. Statement (ii) follows immediately.

To prove part (iii) notice that $A\left( \Delta Y^{c}\right) -A\left( \Delta Y^{n}\right) <0$ under PT. Then, although $\Phi _{\left( \mathrm{iii}\right) }$ is written identically to $\Phi _{\left( \mathrm{ii}\right) }$, its sign is no longer determined. If the term in square brackets is positive, it follows that $\partial X/\partial t\ge 0$ if $\phi _{1t}\le \Phi _{\left( \mathrm{iii}\right) }$ and $\partial X/\partial t<0$ if $\phi _{1t}>\Phi _{\left( \mathrm{iii}\right) }$. However, if the term in brackets is negative, then $\partial X/\partial t\ge 0$ if $\phi _{1t}>\Phi _{\left( \mathrm{iii}\right) }$ and $\partial X/\partial t<0$ if $\phi _{1t}<\Phi _{\left( \mathrm{iii}\right) }$. Finally, notice that the term in square brackets is positive if its first (positive) term outweighs the second. This occurs when $\phi _{1}=0$ (and by continuity, in its neighbourhood) and when *s* tends to ${\bar{s}}$ (for then *X* tends to *Y* and $\left[ A\left( \Delta Y^{c}\right) -A\left( \Delta Y^{n}\right) \right]$ tends to zero).

(v) Under the relevant parameter restrictions, the derivative $\partial X/\partial t$ is

$$\begin{aligned} \frac{\partial X}{\partial t}= & {} \frac{ft+s}{D}\left[ \left[ f\left[ \left[ \phi _{1}-1\right] \left[ Y-X\right] +\phi _{2}\right] +\left[ ft+s\right] \phi _{2t}\right] \left[ \phi _{1}-1\right] w\left( p\right) v^{\prime \prime }\left( \Delta Y^{c}\right) \right. \\&\left. +\left[ f\left[ \phi _{1}\left[ Y-X\right] +\phi _{2}\right] +\left[ ft+s\right] \phi _{2t}\right] \phi _{1}\left[ 1-w\left( p\right) \right] v^{{\prime }{\prime }}\left( \Delta Y^{n}\right) \right] . \end{aligned}$$

Using the first-order condition in (4) and replacing $\varepsilon _{\phi _{2},t}\equiv t\left[ \phi _{2t}/\phi _{2}\right]$, we obtain

$$\begin{aligned} \frac{\partial X}{\partial t}= & {} \frac{f\left[ Y-X\right] }{ft+s}+\frac{\phi _{2}}{t}\left[ tf+\left[ tf+s\right] \varepsilon _{\phi _{2},t}\right] \\&\times \frac{ A\left( \Delta Y^{c}\right) -A\left( \Delta Y^{n}\right) }{\left[ ft+s\right] \left[ \left[ \phi _{1}-1\right] A\left( \Delta Y^{c}\right) -\phi _{1}A\left( \Delta Y^{n}\right) \right] }. \end{aligned}$$

The second-order condition in (5) writes as

A.3 $$\begin{aligned} \frac{\partial ^{2}V}{\left[ \partial X\right] ^{2}}\!=\!\left[ ft\!+\!s\right] ^{2} \left[ w\left( p\right) \left[ 1\!-\!\phi _{1}\right] ^{2}v^{\prime \prime }\left( \Delta Y^{c}\right) \!+\!\left[ 1-w\left( p\right) \right] \left[ \phi _{1}\right] ^{2}v^{\prime \prime }\left( \Delta Y^{n}\right) \right] <0, \nonumber \\ \end{aligned}$$

hence

$$\begin{aligned} \left[ \phi _{1}-1\right] A\left( \Delta Y^{c}\right) -\phi _{1}A\left( \Delta Y^{n}\right) <0. \end{aligned}$$

As also $A\left( \Delta Y^{c}\right) -A\left( \Delta Y^{n}\right) <0$ (under diminishing sensitivity), a sufficient condition for $\partial X/\partial t>0$ is, therefore, $\varepsilon _{\phi _{2},t}<-ft/\left[ ft+s\right]$. $\square$

### Proof of Lemma 3

We first prove the statements relative to $\mathrm{d}\left[ {\mathbf {E}}\left( \Delta Y\right) \right] /\mathrm{d}t$ and then move on to those relative to $\mathrm{d}\left[ \Delta Y^{n}\right] /\mathrm{d}t$. From ${\mathbf {E}}\left( \Delta Y\right) =\left[ ft+s\right] \left[ Y-X\right] \left[ \phi _{1}-w\left( p\right) \right]$ we compute

$$\begin{aligned} \frac{\mathrm{d}{\mathbf {E}}\left( \Delta Y\right) }{\mathrm{d}t}= & {} f\left[ Y-X\right] \left[ \phi _{1}-w\left( p\right) \right] -\left[ ft+s\right] X_{t}\left[ \phi _{1}-w\left( p\right) \right] \\&+\left[ ft+s\right] \left[ Y-X\right] \phi _{1t} =\left[ \phi _{1}-w\left( p\right) \right] \left[ ft+s\right] \\&\times \left[ \frac{ f\left[ Y-X\right] }{\left[ ft+s\right] }-X_{t}\right] +\left[ ft+s\right] \left[ Y-X\right] \phi _{1t}. \end{aligned}$$

Replacing $X_{t}$ by its expression, we obtain $d{\mathbf {E}}\left( \Delta Y\right) /\mathrm{d}t=\Omega \phi _{1t}$ with

$$\begin{aligned} \Omega\equiv & {} \frac{-1}{\left[ \phi _{1}-1\right] A\left( \Delta Y^{c}\right) -\phi _{1}A\left( \Delta Y^{n}\right) } \\&\times \; \left[ \left[ Y-X\right] \left[ ft+s\right] \left[ \begin{array}{c} \left[ 1-w\left( p\right) \right] A\left( \Delta Y^{c}\right) \\ +w\left( p\right) A\left( \Delta Y^{n}\right) \end{array} \right] +\frac{\left[ \phi _{1}-w\left( p\right) \right] \left[ pv^{\prime }\left( \Delta Y^{c}\right) \right] }{\left[ 1-p\right] \left[ \phi _{1} \right] ^{2}v^{\prime }\left( \Delta Y^{n}\right) }\right] . \end{aligned}$$

Studying the sign of $\Omega$, by the second-order condition in (A.3), the initial fraction is positive. Within the main square bracket, the first term is positive under RD while the second term is always positive, for $\phi _{1}>w\left( p\right)$ by Lemma . This proves that under RD $\mathrm{d}\left[ {\mathbf {E}}\left( \Delta Y\right) \right] /\mathrm{d}t\gtreqless 0\Leftrightarrow \phi _{1t}\gtreqless 0$. Under PT, the first term in brackets may take negative values. However, for $s={\overline{s}}$ (and, by continuity, in its neighbourhood), then $Y=X$ and the first term in square bracket is zero. This proves that for *s* sufficiently close to ${\overline{s}}$, $\mathrm{d}\left[ {\mathbf {E}}\left( \Delta Y\right) \right] /\mathrm{d}t\gtreqless 0\Leftrightarrow \phi _{1t}\gtreqless 0$.

Moving to $\Delta Y^{n}$, from $\Delta Y^{n}=\phi _{1}\left[ ft+s\right] \left[ Y-X\right]$ we compute

$$\begin{aligned} \frac{\mathrm{d}\left[ \Delta Y^{n}\right] }{\partial t}= & {} \left[ f\phi _{1}+\left[ tf+s\right] \phi _{1t}\right] \left[ Y-X\right] -\phi _{1}\left[ ft+s\right] X_{t} \\= & {} \left[ ft+s\right] \left[ \begin{array}{c} \left[ Y-X\right] \left[ 1-\frac{\left[ ft+s\right] ^{2}\left[ \phi _{1} \right] ^{2}\left[ 1-w\left( p\right) \right] v^{\prime }\left( \Delta Y^{n}\right) \left[ A\left( \Delta Y^{c}\right) -A\left( \Delta Y^{n}\right) \right] }{D}\right] \\ -\frac{\left[ ft+s\right] }{D}\left[ w\left( p\right) v^{\prime }\left( \Delta Y^{c}\right) \right] \end{array} \right] \phi _{1t}. \end{aligned}$$

Under RD, $A\left( \Delta Y^{c}\right) -A\left( \Delta Y^{n}\right) >0$; hence, all the terms in the bracket are positive. This directly implies that $\mathrm{d}\left[ \Delta Y^{n}\right] /\mathrm{d}t\gtreqless 0\Leftrightarrow \phi _{1t}\gtreqless 0$. Instead, under PT we have $A\left( \Delta Y^{c}\right) -A\left( \Delta Y^{n}\right) <0$. However, for $s={\overline{s}}$ (and, by continuity, in its neighbourhood), then $Y=X$ and the first term in square bracket is zero: $\partial \mathrm{d}\left[ \Delta Y^{n} \right] /\partial t=-\frac{\left[ ft+{\overline{s}}\right] ^{2}}{D}\left[ pv^{\prime }\left( \Delta Y^{c}\right) \right] \phi _{1t}$. This proves that for *s* sufficiently close to ${\overline{s}}$, $\mathrm{d}\left[ \Delta Y^{n}\right] /\mathrm{d}t\gtreqless 0\Leftrightarrow \phi _{1t}\gtreqless 0$. $\square$

### Proof of Lemma 4

Setting $\phi _{2}=0$ the taxpayer’s objective function can be written as

A.4 $$\begin{aligned} V= & {} w\left( p\right) \left[ \left[ tf+s\right] \left[ Y-X\right] \right] ^{\beta ^{1-DS}\gamma ^{DS}}v\left( \phi _{1}-1\right) \nonumber \\&+\left[ 1-w\left( p\right) \right] \left[ tf+s\right] ^{\beta }\left[ Y-X\right] ^{\beta }v\left( \phi _{1}\right) . \end{aligned}$$

Thus, $\partial V/\partial X$ is found as

$$\begin{aligned}&-\left[ tf+s\right] ^{\beta ^{1-DS}\gamma ^{DS}}\left[ Y-X\right] ^{\beta ^{1-DS}\gamma ^{DS}-1}\\&\quad \times \left[ \begin{array}{c} \left[ \beta ^{1-DS}\gamma ^{DS}w\left( p\right) -\left[ Y-X\right] w^{\prime }p^{\prime }\right] v\left( \phi _{1}-1\right) \\ +\left[ \beta ^{1-DS}\gamma ^{DS}\left[ 1-w\left( p\right) \right] +\left[ Y-X\right] w^{\prime }p^{\prime }\right] v\left( \phi _{1}\right) \end{array} \right] . \end{aligned}$$

If $\phi _{1}\ge 1$ then $\partial V/\partial X<0$ and if $\phi _{1}\le 0$ then $\partial V/\partial X>0$. In either case, there cannot be an interior maximum. Hence, at an interior maximum $\phi _{1}\in \left( 0,1\right)$. $\square$

### Proof of Proposition 2

(i) Under $\mathrm{LA}\wedge \mathrm{PW}$ the first-order condition is written, using A9, as

A.5 $$\begin{aligned} \frac{\partial V}{\partial X}= & {} w\left( p\right) \left\{ t\left[ f-1\right] +s\right\} v^{\prime }\left( Y^{c}\right) -\left[ 1-w\left( p\right) \right] tv^{\prime }\left( Y^{n}\right) \nonumber \\&+w^{\prime }p^{\prime }\left[ v\left( Y^{c}\right) -v\left( Y^{n}\right) \right] =0. \end{aligned}$$

Thus, $\partial X/\partial t$ is found as

$$\begin{aligned} \frac{\partial X}{\partial t}=-\frac{1}{\beta D}\left\{ \begin{array}{c} \left[ \beta w\left( p\right) \left[ f-1\right] -w^{\prime }p^{\prime }\left[ X+f\left[ Y-X\right] \right] \right] v^{\prime }\left( Y^{c}\right) \\ -\left[ \beta \left[ 1-w\left( p\right) \right] -w^{\prime }p^{\prime }X \right] v^{\prime }\left( Y^{n}\right) \\ -\left[ \beta w\left( p\right) \left\{ t\left[ f-1\right] +s\right\} +w^{\prime }p^{\prime }Y^{c}\right] \left[ X+f\left[ Y-X\right] \right] v^{\prime \prime }\left( Y^{c}\right) \\ +\left[ \beta t\left[ 1-w\left( p\right) \right] +w^{\prime }p^{\prime }Y^{n} \right] Xv^{\prime \prime }\left( Y^{n}\right) \end{array} \right\} . \end{aligned}$$

If $s=0$, and noting that $A\left( Y^{i}\right) =\left[ 1-\beta \right] /Y^{i}$, $i=c,n$, $\partial X/\partial t$ reduces to

A.6 $$\begin{aligned} \frac{\partial X}{\partial t}=-\frac{Y}{\beta tD}\left\{ \begin{array}{c} w^{\prime }p^{\prime }\left[ v^{\prime }\left( Y^{n}\right) -v^{\prime }\left( Y^{c}\right) +\left[ 1-\beta \right] \left[ \frac{Y^{n}-Y^{c}}{Y^{n}} \right] v^{\prime }\left( Y^{c}\right) \right] \\ +\beta tw\left( p\right) \left[ f-1\right] \left[ 1-\beta \right] \left[ \frac{Y^{n}-Y^{c}}{Y^{n}Y^{c}}\right] v^{\prime }\left( Y^{c}\right) \end{array} \right\} . \end{aligned}$$

Now note that from the first-order condition and A9 we have

$$\begin{aligned} \frac{\beta tw\left( p\right) \left[ f-1\right] +w^{\prime }p^{\prime }Y^{c} }{\beta t\left[ 1-w\left( p\right) \right] +w^{\prime }p^{\prime }Y^{n}}= \frac{v^{\prime }\left( Y^{n}\right) }{v^{\prime }\left( Y^{c}\right) }= \left[ \frac{Y^{c}}{Y^{n}}\right] ^{1-\beta }. \end{aligned}$$

Then, defining $\phi \left( z\right) =z^{1-\beta }$, the first-order Taylor series expansion of $\phi \left( z\right)$ at $z=1$ is $g\left( z\right) =1-\left[ 1-\beta \right] \left[ 1-z\right]$. Clearly, $g(z)\ge \phi \left( z\right)$ for all *z* and $g(z)>z^{1-\beta }$ for all $z\ne 1$, so

$$\begin{aligned} \frac{v^{\prime }\left( Y^{n}\right) }{v^{\prime }\left( Y^{c}\right) } <g\left( \frac{Y^{c}}{Y^{n}}\right) =1-\left[ 1-\beta \right] \left[ \frac{ Y^{n}-Y^{c}}{Y^{n}}\right] . \end{aligned}$$

Rearranging, we obtain

A.7 $$\begin{aligned} v^{\prime }\left( Y^{n}\right) -v^{\prime }\left( Y^{c}\right) +\left[ 1-\beta \right] \left[ \frac{Y^{n}-Y^{c}}{Y^{n}}\right] v^{\prime }\left( Y^{c}\right) <0, \end{aligned}$$

which signs (A.6) positive. As Eq. (A.6) holds for $w\left( p\right) =p$, and it is independent of $\lambda$, $\partial X/\partial t\ge 0$ also holds under EUT. If $s\uparrow {\bar{s}}$ then $\partial X/\partial t$ instead writes as

$$\begin{aligned} \frac{\partial X}{\partial t}=\lim _{s\uparrow {\bar{s}}}\frac{w\left( p\right) s}{tD}v^{\prime }\left( Y^{c}\right) <0\text {.} \end{aligned}$$

(ii)–(iii) Setting $\phi _{2}=0$ the taxpayer’s objective function is given by (A.4). Using the first order condition, we obtain

$$\begin{aligned} \frac{\partial X}{\partial t}= & {} \frac{\left[ tf+s\right] ^{\beta ^{1-DS}\gamma ^{DS}}\left[ Y-X\right] ^{\beta ^{1-DS}\gamma ^{DS}-1}}{D} \\&\times \;\left[ \beta ^{1-DS}\gamma ^{DS}w\left( p\right) -\left[ Y-X\right] w^{\prime }p^{\prime }\right] v\left( \phi _{1}-1\right) \\&\times \;\left[ f\left[ \beta ^{1-DS}\gamma ^{DS}-\beta \right] +\phi _{1t} \left[ ft+s\right] \frac{\beta ^{1-DS}\gamma ^{DS}\phi _{1}-\beta \left[ \phi _{1}-1\right] }{\phi _{1}\left[ \phi _{1}-1\right] }\right] . \end{aligned}$$

Under PT ($DS=1$) we, therefore, obtain

A.8 $$\begin{aligned} \frac{\partial X}{\partial t}=\frac{\left[ tf+s\right] ^{\beta }\left[ Y-X \right] ^{\beta -1}}{\phi _{1}D}\phi _{1t}\left[ \beta w\left( p\right) - \left[ Y-X\right] w^{\prime }p^{\prime }\right] v\left( \phi _{1}-1\right) , \end{aligned}$$

which takes the sign of $-\phi _{1t}$. Under RD ($DS=0$) we obtain

$$\begin{aligned} \frac{\partial X}{\partial t}\gtreqqless 0\Leftrightarrow \phi _{1t}\lesseqqgtr \frac{f\left[ \gamma -\beta \right] \phi _{1}\left[ \phi _{1}-1\right] }{\left[ tf+s\right] \left[ \gamma \phi _{1}-\beta \left[ \phi _{1}-1\right] \right] }<0. \end{aligned}$$

$$\square $$

## Footnotes

1 See Feinstein (1991), however, for evidence consistent with a negative relationship.

2 PT was initially proposed by Kahneman and Tversky (1979), and subsequently extended to “cumulative” PT by Tversky and Kahneman (1992). In this study, we use cumulative PT, but our main qualitative conclusions apply also to the original version of PT. See, e.g., Barberis (2013) and Camerer (2000) for reviews of applications of PT other than to tax evasion.

3 Among others, the TBM allows us to treat as special cases the models developed by Bernasconi and Zanardi (2004), Dhami and al-Nowaihi (2007) and two cases described in Hashimzade et al. (2013). Our model can be used to reconcile the apparently contradictory results therein. Although we have strived for widespread applicability, the TBM does not exhaustively encompass the literature. In particular, Rablen (2010) introduces PT into a version of the tax evasion model that allows for taxes to fund the provision of a public good (which we do not consider here). In addition, Bernasconi et al. (2014) allow for reference income to adapt over time to changes in the tax rate. These authors show that their model predicts an upward drift in tax evasion (after an initial fall), following an increase in the tax rate.

4 Following the study of Kleven et al. (2011)—who show that the descriptive validity of the EUT model is improved when allowing for plausible levels of third-party reporting—it is now common to interpret *Y* not as a taxpayer’s full income, but as that part of income not subject to third-party reporting. As this interpretation is not adopted, however, in the literature we address, we do not formally adopt this interpretation.

5 Hence, the objective probability distribution is $\left\{ p,1-p\right\}$ and the transformed probability distribution is $\left\{ w\left( p\right) ,1-w\left( p\right) \right\}$. PT allows for different weighting functions to apply to outcomes that fall above or below the reference level. As pointed out by Dhami and al-Nowaihi (2007) and Prelec (1998), however, empirically the same weighting function is found to apply above and below the reference level, so we assume there to be a single weighting function *w* . The assumption $p^{\prime }\le 0$ is consistent with the literature on optimal auditing (e.g., Reinganum and Wilde 1986; Piolatto and Trotin 2016).

6 An example of a function satisfying A0-A4 is $u\left( x\right) =x-e^{-bx}$ with $b>0$. See the study by Bell and Fishburn (2000) for an axiomatisation of functions of this type.

7 These properties continue to hold if $\lambda >1$, the only difference between cases being that, if $\lambda >1$ then, even if $u\left( x\right)$ is differentiable at $x=0$, $v\left( x\right)$ is not differentiable at $x=0$.

8 Local maxima may also arise, so the first-order condition may not possess a unique solution. See the study by Hashimzade et al. (2013) for a detailed discussion of these difficulties.

9 We do not investigate the properties of the corner solutions $X\in \left\{ 0,Y\right\}$, for the descriptive validity of tax evasion as an all-or-nothing activity appears weak.

10
Fisman and Wei (2004) empirically document an instance in which tax evasion indeed leads to a Laffer curve relationship holding. Clearly, however, were the government operating on the wrong side of a Laffer curve, it is unclear why it would ever wish to further increase the tax rate.

11
Trotin (2012) uses the taxpayer’s legal post-tax income to specify reference income in a model without stigma and claims (her Proposition 8) to resolve the Yitzhaki puzzle. The difference between our findings and hers is attributable to a non-sequitur in her proof of Proposition 8. In particular, we are unable to replicate the expression for $\partial \Phi _{{\overline{R}} }\left( x^{*},t\right) /\partial t$ in the first line of her proof.

12 Consistent with our finding regarding probability weighting, Eide (2001) shows that introducing (rank-dependent) probability weighting into the standard tax evasion model does not qualitatively change the comparative statics results.

13 An example $u\left( x\right)$ that satisfies A0–A4 is given by the piecewise function $u\left( x\right) =ax^{\beta }$ for $x\ge 0$ and $u\left( x\right) =ax^{1/\beta }$ for $x<0$, where $a>0$ and $\beta \in \left( 0,1\right)$.

14 We have shown (Table 1) that the specification of reference income in the study by Dhami and al-Nowaihi (2007) corresponds to a convex combination of $$Y^{c}$$ and $$ Y^{n}$$ with a weight on $$Y^{c}$$ of $$\alpha =\phi _{1}=t/\left[ ft+s\right] $$ . A possible confusion in evaluating Dhami and al-Nowaihi’s result, which our analysis enables us to clarify, is that these authors assert this specification to be the only one with the property $$R\in (Y^{c},Y^{n})$$. We show, however, that this specification is just one special case of a wider set of specifications with this property. In particular, any specification of reference income as a (strictly) convex combination of $$Y^{c}$$ and $$Y^{n}$$ must also satisfy this property (Lemma 2).

15 The specification of the value and weighting functions of PT may also be problematic (see, e.g., Neilson and Stowe 2002; Sadiraj 2014).
